# Evaluation of Pathogenetic and Immunological Parameters of the Genotype I and II Recombinant African Swine Fever Viruses Detected in Pigs in Vietnam

**DOI:** 10.3390/v18060635

**Published:** 2026-05-31

**Authors:** Anh Duc Truong, Hien Thi Thu Nguyen, Nhu Thi Chu, Linh Phuong Nguyen, Khanh Quoc Dam, Le Thi Hai Vo, Tuong Dinh Nguyen, Sun A. Choi, Seon Hoe Kim, Jung Hee Lee, Seong Cheol Moon, Jung Hyang Sur, Ha Thi Thanh Tran, Hoang Vu Dang

**Affiliations:** 1 Department of Veterinary Immunology and Epidemiology, Vietnam Institute of Animal and Veterinary Sciences (VIAVS), 86 Truong Chinh, Kim Lien Da, Hanoi 100000, Vietnam; truonganhduc84@gmail.com (A.D.T.); nguyenthithuhien@naue.edu.vn (H.T.T.N.); chunhuk58tyc@gmail.com (N.T.C.); ngphuonglinhwhale2811@gmail.com (L.P.N.); damkhanh699@gmail.com (K.Q.D.); 2 Faculty of Agriculture, Forestry and Fisheries, Nghe An University, 51 Ly Tu Trong, Vinh City 460000, Nghe An, Vietnam; levth@nau.edu.vn (L.T.H.V.); tuongnd@nau.edu.vn (T.D.N.); 3 Central Research and Development Institute, Komipharm International Co., Ltd., 17 Gyeongje-ro, Siheung-si 15094, Gyeonggi-do, Republic of Korea; dktjschl@komipharm.com (S.A.C.); seonhwi12@komipharm.com (S.H.K.); heeya8421@komipharm.com (J.H.L.); moonvet@komipharm.com (S.C.M.); surpathol@komipharm.com (J.H.S.)

**Keywords:** ASFV, cytokine, clinical sign, immune response, recombinant genotype

## Abstract

Recombinant genotype I–II African swine fever virus (ASFV) strains with high virulence have been increasingly reported in China and Vietnam since 2023, raising significant concerns for disease control. In this study, we characterized the hematological, virological, pathological, and immunological dynamics in ASFV-inoculated pigs, with particular emphasis on temporal changes associated with mortality following the onset of viremia. Specific-pathogen-free pigs were intramuscularly inoculated with 1 × 10^3^ or 1 × 10^5^ HAD_50_/mL of ASFV LS100 virus strain and developed acute disease characterized by high fever and severe hemorrhagic manifestations. The incubation period ranged from 3 to 5 days, with mortality occurring between 6 and 10 days post-inoculation (dpi). Viral genomic DNA was detected in blood, oral swabs, and rectal swabs as early as 2–4 dpi. Pathological examination revealed prominent necrotic skin lesions and joint swelling. Although hematological parameters and serum biochemical profiles were comparable between high- and low-dose groups, differences in viral load distribution were observed. Notably, cytokine profiling in whole blood revealed a strong and persistent upregulation of pro-inflammatory mediators, including IL-1β, IL-6, IL-12p40, TNF-α, IFN-γ, CCL2, CCL3, CCL14, CXCL9, and CXCL10, which correlated with persistent fever from 2 to 7 dpi. Collectively, these findings confirm that naturally occurring recombinant genotype I–II ASFV strains are highly virulent and capable of inducing severe systemic inflammation. Their continued circulation poses substantial challenges for ASF control and prevention in Vietnam and threatens the global swine industry.

## 1. Introduction

African swine fever (ASF) is a highly contagious and devastating viral disease of swine, posing a serious threat to the global pig industry [1]. The causative agent, African swine fever virus (ASFV), belongs to the genus *Asfivirus* within the family *Asfarviridae* [1,2]. The B646L gene of ASFV encodes the major capsid protein p72, which plays a critical role in virion assembly and viral attachment to host cells [3,4]. ASFV isolates are classified into 24 genotypes based on the C-terminal region of the B646L gene, showing 86.2–99.5% nucleotide identity [2]. All 24 genotypes have been identified in sub-Saharan Africa, although only two have spread beyond the continent [5,6,7].

In 1957, genotype I ASFV was introduced into Europe, causing outbreaks in several European countries [8]. Subsequently, in 2007, a highly virulent genotype II ASFV (Georgia07) emerged in Georgia, and following its emergence in Georgia in 2007, genotype II ASFV has disseminated widely across Eastern Europe and Central Europe, becoming endemic in multiple wild boar and domestic pig populations. The virus subsequently spread throughout Asia, causing devastating losses in major pork-producing countries. More recently, ASFV genotype II has been reported in the Caribbean, including outbreaks in the Dominican Republic and Haiti, marking its re-emergence in the Americas after several decades of absence. This expanding geographic distribution underscores the persistent risk of transboundary spread and highlights the urgent need for improved surveillance, biosecurity, and effective vaccine strategies on a global scale [9]. By 2018, Georgia07-like ASFVs had reached China and subsequently other Asian countries, including Vietnam (2019), Korea, and Thailand, leading to the loss of more than seven million pigs across Eurasia [10,11,12,13,14]. Currently, no commercial vaccine is available for ASF, and the lack of effective control strategies has allowed the virus to remain endemic and continue evolving.

In 2021, low-virulence genotype I ASFVs were detected in pigs in China [15]. These strains were hemadsorption (HAD)-negative and genetically similar to the NH/P68 strain reported in Portugal in the 1960s. NH/P68 is a genotype I ASFV strain that has been extensively characterized as an attenuated isolate and is widely used as a reference candidate in ASFV vaccine development studies. This strain typically induces mild or subclinical infection while eliciting protective immune responses, making it a valuable model for investigating attenuation and host immunity [5,6,7]. By 2023, recombinant ASFVs containing genomic material from both genotypes I and II were reported and fully characterized in China [16]. Later that year, similar recombinant genotype I–II ASFVs were detected in Vietnam [17]; however, their pathogenicity and immune evasion capabilities had not yet been evaluated in pigs in Vietnam.

In the present study, we isolated recombinant genotype I–II ASFVs from field samples collected in Vietnam. These isolates were characterized, and their virulence and ability to evade host immune responses were evaluated in experimentally infected pigs. The virulence, clinical progression, viral kinetics, and host immune responses were systematically evaluated. The results demonstrate that these recombinant viruses retain a highly virulent phenotype and exhibit strong modulation of host immune responses, underscoring the ongoing evolution of ASFV through inter-genotypic recombination and the resulting challenges for disease control and vaccine development.

## 2. Materials and Methods

### 2.1. Cell Culture and Virus Isolation

Primary porcine alveolar macrophages (PAMs) were isolated from the lungs of 20–30-day-old specific-pathogen-free (SPF) pigs. The cells were maintained in RPMI-1640 medium (Thermo Scientific, Waltham, MA, USA) supplemented with 10% fetal bovine serum (FBS) at 37 °C in a humidified atmosphere containing 5% CO_2_. Homogenates of pig spleen samples collected from the field tested positive for virus by quantitative PCR (qPCR). Virus isolation was performed in PAM cell and combination with the haemadsorption (HAD) assay. For virus isolation, cell supernatants were harvested five days post-inoculation. According to the recommendations of the World Organization for Animal Health, the presence of the ASFV in the supernatant was verified by qPCR targeting the viral p72 gene, alongside the HAD assay to identify infectious particles [1]. Virus stocks were aliquoted and stored at −80 °C. Each stock was further examined to ensure the absence of bacterial contamination and other porcine viruses, including classical swine fever virus (CSFV), porcine reproductive and respiratory syndrome virus (PRRSV), pseudorabies virus (PRV), and porcine circovirus type 2 (PCV2) by Real-time PCR or Real-time RT-PCR.

### 2.2. Viral Genome Detection by qPCR

African swine fever virus (ASFV) genomic DNA was extracted from cell supernatants, tissue homogenates, swabs, or EDTA-treated whole blood using the QIAamp DNA Extraction Kit (Qiagen, Hilden, Germany). Quantitative PCR was conducted using a QuantStudio 5 System (Applied Biosystems, Waltham, MA, USA) in accordance with the WOAH-recommended protocol described by King et al. (2003) [18].

### 2.3. Virus and HAD Assay

The ASFV NIVR-LS08, NIVR-LS100, and NIVR-LS102 virus strains were isolated from the spleen of a pig that died in Lang Son province of Vietnam in 2025. Sequences of the p72 and p54 genes of ASFV strains in this study and reference strains were retrieved from the NCBI GenBank database. Phylogenetic trees were constructed using the neighbor-joining method implemented in MEGA version 7 software (https://www.megasoftware.net/), with 1000 bootstrap replicates. Numbers along the branches indicate bootstrap support values. Red and blue circles indicate ASFV isolates obtained in this study. Scale bars represent the number of nucleotide substitutions per site.

The HAD assay was conducted as described previously by Tran et al. (2020) [19]. Briefly, PAM cells were plated in 96-well plates, after which samples were applied and serially diluted tenfold in triplicate. The ASFV titers were determined by the presence of characteristic rosette formations, indicative of erythrocyte haemadsorption surrounding infected cells. HAD reactions were monitored over a five-day period, and the 50% HAD dose (HAD_50_) was calculated according to the Reed and Muench method [20].

### 2.4. Animal Experiment

To evaluate the virulence of ASFV in pigs, 7-week-old SPF Landrace and Landrace-cross pigs, free of PRRSV, PCV2, PRV, and CSFV, were obtained from farms in Hanoi, Vietnam. Pigs were randomly divided into three groups (five pigs per group) and intramuscularly inoculated with 1 mL of ASFV strain NIVR-LS100 (National Institute of Veterinary Research) at titers of 1 × 10^5^ HAD_50_/mL or 1 × 10^3^ HAD_50_/mL per pig. Pigs were monitored daily for clinical signs, including fever, anorexia, depression, respiratory distress, and hemorrhagic manifestations. Animals reaching severe clinical conditions—such as persistent high fever, recumbency, or inability to access feed and water—were humanely euthanized to minimize suffering. Oral and rectal swabs, as well as whole blood treated with EDTA or heparin, were daily collected for hematology, biochemical analysis, and viral DNA quantification by qPCR at the indicated time points. After collection, swabs were immediately placed into tubes containing viral transport medium, vortexed to release material, and stored at −80 °C until processing. Viral DNA was subsequently extracted from the swab suspensions using the same protocol as for blood and tissue samples and analyzed by qPCR.

Organs and lymph nodes were collected from deceased or euthanized pigs for viral DNA detection using qPCR. Necropsies were performed immediately after death. Tissue samples, including heart, lung, kidney, spleen, liver, stomach, testis, and lymph nodes (intestinal, inguinal, submaxillary, bronchial, gastrohepatic, and mediastinal), were collected during necropsy. Viral DNA was detected in these samples using qPCR, and gross lesions were evaluated. Clinical signs were scored as described previously, with minor modifications (Supplementary Table S1).

### 2.5. Blood Count and Biochemical Analysis

Blood samples were collected daily from the jugular vein of each pig before morning feeding. Samples were placed into HTM K_2_EDTA tubes (HTM, Ho Chi Minh, Vietnam) containing 2 mL of anticoagulant solution (EDTA) or lithium heparin tubes (HTM, Vietnam). Complete blood counts—including white blood cells (WBCs), red blood cells (RBCs), hemoglobin, lymphocytes, and platelet counts—were performed using an SMT-120VP automated hematology analyzer (Chengdu Seamaly Technology Co., Ltd., Hezuo Road, Hi-tech Zone, Chengdu, China). Results were compared with the manufacturer’s reference values for pigs. Serum was separated by centrifugation at 1800× *g* for 10 min and stored at −20 °C. Biochemical analyses were conducted using a semi-automatic biochemical analyzer (SMT-120VP, Chengdu Seamaly Technology Co., Ltd., China). Parameters, including alanine aminotransferase (ALT), aspartate aminotransferase (AST), creatinine (CREA), urea (UREA), and albumin (ALB), were measured to assess potential liver and kidney damage. All biochemical analyses were performed at Pet One Clinic, Hanoi, Vietnam.

### 2.6. RNA Extraction, cDNA Synthesis, and qPCR

Total RNA was isolated from whole blood using TRIzol reagent (Invitrogen, Carlsbad, CA, USA). Up to 2 µg of total RNA was treated with 1.0 U DNase I and 1.0 µL of 10× reaction buffer (Thermo Fisher Scientific, Waltham, MA, USA) at 37 °C for 30 min. The reaction was terminated by adding 1.0 µL of 50 mM EDTA and heating at 65 °C for 10 min.

Complementary DNA (cDNA) was synthesized using the Maxima First Strand cDNA Synthesis Kit (Thermo Fisher Scientific) following the manufacturer’s instructions. Gene expression levels of cytokines and chemokines were quantified by RT-qPCR using gene-specific primers (Table 1) and 2× Ampigen SYBR Green Master Mix (Enzo Life Sciences, Farmingdale, NY, USA) on a QuantStudio 5 Real-Time PCR System (Thermo Fisher Scientific, Waltham, MA, USA). Glyceraldehyde-3-phosphate dehydrogenase (GAPDH) was used as an internal reference gene. Relative gene expression was calculated using the 2^−ΔΔCt^ method after normalization to GAPDH expression [21].

### 2.7. Statistical Analysis

All data are expressed as mean ± standard error of the mean (SEM) from at least three replicates. Statistical analyses were conducted using IBM SPSS software (version 25.0 for Windows; IBM Corp., Chicago, IL, USA). Differences between groups were analyzed using Duncan’s multiple comparison test, and *p* < 0.05 was considered statistically significant.

## 3. Results

### 3.1. Isolation and Sequence Analysis of the ASFV Isolates

**Figure 1:**
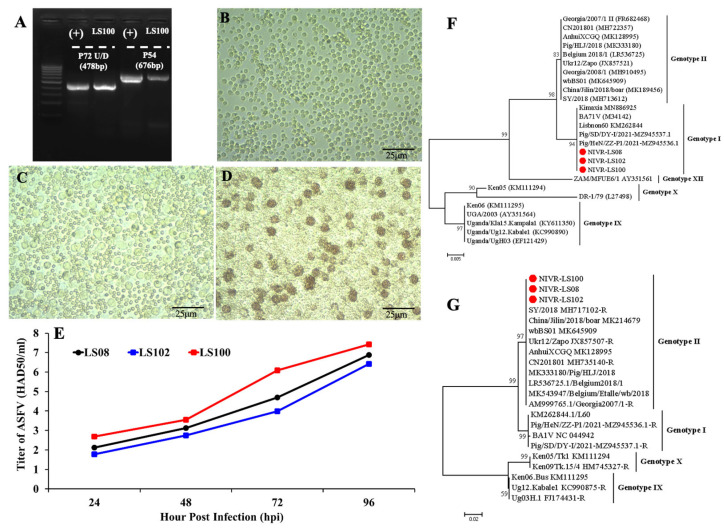
Detection of ASFV in a field sample of spleen tissue. (**A**) PCR detection of p72 (478bp) and p54 (676bp) genes in spleen DNA from the suspected pig; (+) Positive control and LS100 as the sample in this study. (**B**) PAM-negative cell without pig blood cells; (**C**) PAM-negative cell with pig blood cells; (**D**) HAD assay of the spleen homogenate. The 10-times dilution of the homogenate supernatant was inoculated into PAM cells with 1% pig blood cells. HAD was observed for 5 days, (original magnification, 400×). (**E**) Growth kinetics of ASFV isolates in PAM cells. PAM cell monolayers were infected with equal amounts of virus at an MOI of 0.01. Cell supernatants were harvested at 24, 48, 72, and 96 h post-inoculation (hpi) and titrated in PAM cells. Data are presented as mean virus titers ± SD (log_10_ HAD_50_/mL), red color for ASFV LS100, black color represented for ASFV LS08 and blue color for ASFV LS102. Phylogenetic analysis of ASFV based on its partial p72 (**F**) and p54 (**G**) gene. The sequences of the p72 or p54 genes of representative ASFVs were downloaded from the NCBI database. The neighbor-joining method was used to construct phylogenetic trees using MEGA7 software. Numbers along branches indicate 1000 bootstraps. The red circles indicate the ASFV isolates from this study. Scale bars indicate nucleotide substitutions per site.

We performed real-time PCR targeting the p72 gene sequence to confirm that the field spleen samples are positive for ASFV, as recommended by WOAH. On the other hand, we also used the real-time PCR targeting the p72 and p54 genes to confirm the genotype of ASFV from the field. The positive 478 bp product of the p72 gene and the 676 bp product of the p54 gene were obtained (Figure 1A). On day two post-inoculation with the supernatant of the spleen homogenate, primary PAMs showed clear HAD, even in the wells inoculated with a 100-fold diluted sample (Figure 1D) compared with a negative control without red blood cells (Figure 1B) and with 1% red blood cells (Figure 1C). These results confirmed that the spleen homogenate contained infectious ASFV.

Sequence analysis of p72 suggested that three ASFV strains, including NIVR-LS100, NIVR-LS102, and NIVR-LS08, share a high identity with SD/DI-J/2021 (GenBank: MZ945537), HeN/ZZ-P1/2021 (GenBank: MZ945536) causing the first ASFV genotype I in China during 2021 [16] and with the ASFV strains HeN/123014/22 (GenBank: OQ504954), IM/DQDM/22 (GenBank: OQ504955), and JS/LG/21 (GenBank: OQ504956) that caused the first ASFV recombinant genotype I and II in China during 2023 [16]. Phylogenetic analysis based on partial p72 genes showed that three ASFV strains, including NIVR-LS100, NIVR-LS102, and NIVR-LS08, belong to Genotype I and have a genetically close relationship with the ASFV strains in China (Figure 1F). However, the sequence analysis of the p54 gene indicated that three ASFV strains, including NIVR-LS100, NIVR-LS102, and NIVR-LS104, have the highest similarity with the sequence of an ASFV strains HeN/123014/22 (GenBank: OQ504954), IM/DQDM/22 (GenBank: OQ504955), and JS/LG/21 (GenBank: OQ504956) that caused the first ASFV recombinant genotype I and II in China, 2023 and low similarity with SD/DI-J/2021 (GenBank: MZ945537), HeN/ZZ-P1/2021 (GenBank: MZ945536) causing the first ASFV genotype I in China, 2023. Phylogenetic analysis based on partial p54 genes showed that three ASFV strains, including NIVR-LS100, NIVR-LS102, and NIVR-LS08, belong to Genotype II and have a genetically close relationship with the ASFV strains in China (Figure 1G). The whole genome sequencing of NIVR-LS08, NIVR-LS100, and NIVR-LS102 strains was sequenced and deposited at GenBank with Acc. LC921668, LC921665, and LC921673, respectively; furthermore, phylogenetic analysis indicated that it belongs to recombinant ASFV genotype I/II. These results demonstrate that the three emerging viruses are complicated recombinants of genotype I and genotype II ASFVs in Vietnam.

To assess the in vitro replication capability of ASFV recombinant genotype I/II isolates, the growth kinetics of NIVR-LS100, NIVR-LS102, and NIVR-LS08 were analyzed in PAM cells and calculated HAD_50_/mL. The results demonstrated that the ASFV NIVR-LS100, NIVR-LS102, and NIVR-LS08 isolates in this study are replicated efficiently in PAM cells. Thus, virus replication peaked between 72 and 96 h post-inoculation (hpi), with average peak tiers ranging from 6.4 to 7.4 log10 HAD_50_/mL, in which the ASFV NIVR-LS100 had a higher titer than other virus strains, up to 7.4 HAD_50_/mL at 96 hpi, and we selected ASFV NIVR-LS100 for further study (Figure 1E).

### 3.2. The Virulence of ASFV NIVR-LS100 in Domestic Pigs

**Figure 2:**
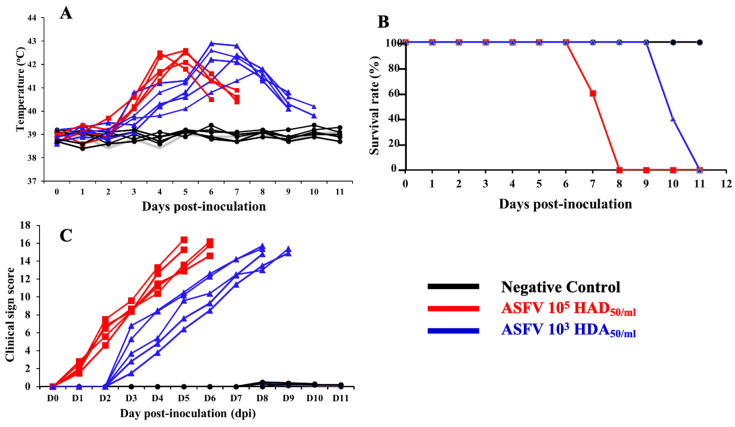
Pathogenicity of the recombinant African swine fever virus NIVR-LS100 in pigs. Groups of five SPF pigs were inoculated with 1 × 10^5^ HAD_50_/mL (red line) or 1 × 10^3^ HAD_50_/mL (blue line) of NIVR-LS100. Black line—control group and dash line as reference interval. The rectal temperature (**A**), survival of the pigs (**B**), and clinical sign scores (**C**) were monitored daily.

Pigs infected with 1 × 10^5^ HAD_50_/mL of ASFV NIVR-LS100 developed a rapid and severe clinical course, with onset of fever (>40.5 °C) by 3 dpi and uniformly high temperatures (41–42.5 °C) at 4–6 dpi, culminating in death at 6–7 dpi (Figure 2A,B). Clinical signs included anorexia, depression, lethargy, cutaneous hyperemia, cyanosis of extremities, respiratory distress, and occasional vomiting. In contrast, pigs infected with 1 × 10^3^ HAD_50_/mL showed a delayed disease progression, with fever appearing at 4–5 dpi and peaking at 6–7 dpi (40.5–43.0 °C), followed by a decline prior to death at 9–10 dpi. Clinical signs in this group were similar but developed later and progressed more gradually. No fever or clinical abnormalities were observed in the control group (Figure 2A,B).

Pigs infected with 1 × 10^5^ HAD_50_/mL of ASFV NIVR-LS100 developed typical clinical signs, including high fever, anorexia, lethargy, respiratory distress, and cutaneous petechiation beginning at 3–5 dpi, whereas pigs infected with 1 × 10^3^ HAD_50_/mL showed a similar but delayed progression (by 1–2 days) (Figure 2C). All pigs in the high-dose group reached severe clinical scores (>10) by 5 dpi and died at 6–7 dpi, while the low-dose group reached similar severity after 7 dpi and died at 9–10 dpi. Gross pathological examination revealed consistent, systemic lesions dominated by widespread congestion and hemorrhage, particularly affecting the spleen, lymph nodes, lungs, kidneys, liver, stomach, and mesenteric tissues (Supplementary Figure S1 and Table S2). These findings indicate a severe, acute hemorrhagic disease consistent with highly virulent ASFV infection.

### 3.3. Replication and Shedding of ASFV NIVR-LS100 in Pigs

Necropsy of all pigs infected with 1 × 10^5^ and 1 × 10^3^ HAD_50_/mL of ASFV NIVR-LS100 revealed consistent gross lesions (Supplementary Figure S1 and Table S2). The most prominent findings were systemic congestion and hemorrhage, particularly in lymphoid organs. All pigs exhibited cyanosis of the ears and markedly enlarged, congested, and friable lymph nodes. Splenomegaly with congestion, necrosis, and hemorrhage, as well as swollen and hemorrhagic tonsils, were observed in all animals. Gastrointestinal involvement was common, with intestinal hemorrhage detected in all pigs in the high-dose group and in most pigs in the low-dose group. Renal hemorrhage was less frequent, occurring in a subset of animals. Additional lesions, including pulmonary edema, hepatic congestion/hemorrhage, hydropericardium, gastric mucosal hemorrhages, and testicular congestion, were observed variably across animals. Overall, the lesions were consistent with an acute, systemic hemorrhagic disease typical of highly virulent ASFV infection.

**Figure 3:**
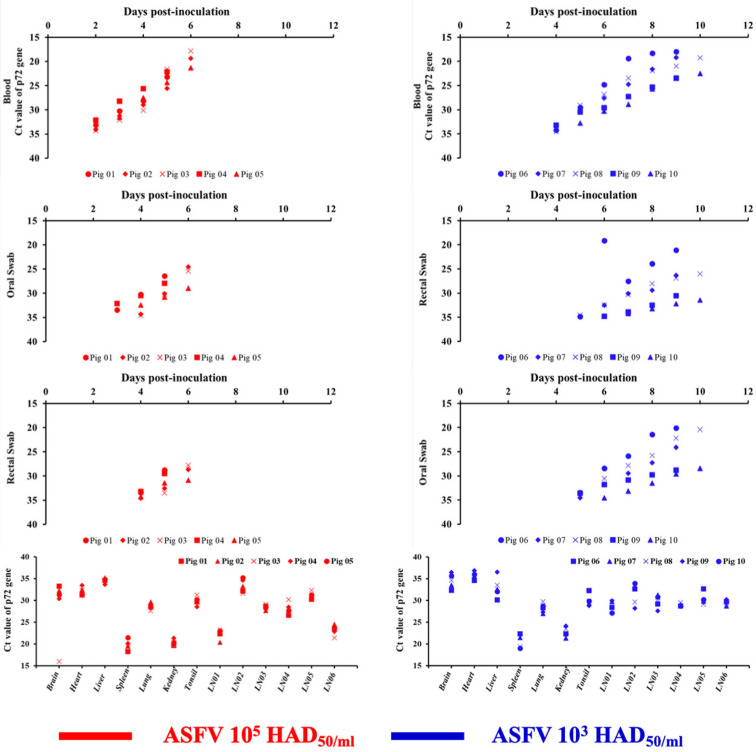
Detection of virus shedding and viremia infection using qPCR. At the indicated days post-infection, oral and rectal swab samples, blood, and tissues were collected from pigs infected with recombinant ASFV NIVR-LS100. Viral DNA was extracted and detected by using qPCR. The data on the pigs cohoused with the 1 × 10^5^ HAD50-inoculated pigs and 1 × 10^3^ HAD50-inoculated pigs are labeled in red and blue, respectively. The differently shaped black dots represent individual pigs. LN1, intestinal lymph node; LN2, inguinal lymph node; LN3, submaxillary lymph node; LN4, bronchial lymph node; LN5, gastrohepatic lymph node; and LN6, mediastinal lymph node.

Blood, oral swab, and rectal swab samples were collected from pigs infected with ASFV and analyzed by qPCR targeting the p72 gene. In the pigs infected with 1 × 10^5^ HAD_50_/mL of ASFV NIVR-LS100 strain, the viral genomic DNA became detectable from blood on 2–3 dpi (Cq values ranged from 32.12 to 34.24), from the oral swabs on 3–4 dpi, and from the rectal swabs on 4–5 dpi (Figure 3). Detection of viral genomic DNA in pigs inoculated with 1 × 10^5^ HAD_50_/mL occurred two days earlier than in those inoculated with 1 × 10^3^ HAD_50_/mL (Figure 3). Tissue specimens, including brain, heart, liver, spleen, lung, kidney, tonsil, and multiple lymph nodes (intestinal, inguinal, submaxillary, bronchial, gastrohepatic, and mediastinal), were collected from all pigs for qPCR analysis. Viral genomic DNA was detected in all examined tissues, with the spleen, kidney, and lymph nodes generally exhibiting higher viral loads compared to other organs at both doses of NIVR-LS100 infection (Figure 3).

### 3.4. Blood Counts

**Figure 4:**
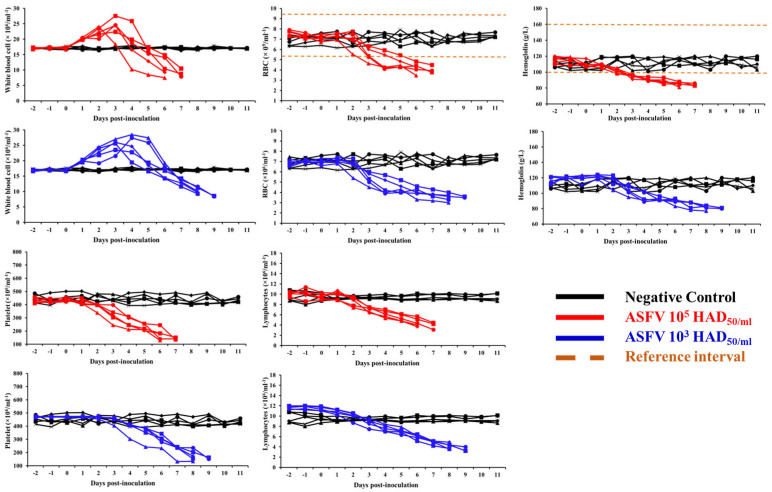
Blood counts during infection: white blood cells, red blood cells, hemoglobin, platelets, and lymphocytes. Groups of five SPF pigs were inoculated with 1 × 10^5^ HAD_50_/mL (red line) or 1 × 10^3^ HAD_50_/mL (blue line) of NIVR-LS100. Black line—control group. The reference intervals of each blood parameter are represented as dotted lines.

Hematological analysis revealed consistent, dose-dependent trends associated with acute ASFV infection. In both groups, changes in white blood cell (WBC) counts were first detected at 2–3 dpi in the 1 × 10^5^ HAD_50_/mL group and at 3–4 dpi in the 1 × 10^3^ HAD_50_/mL group. An initial transient increase in WBCs was observed during the early stage of infection, followed by a progressive decline over time. By the late stage, WBC counts decreased to 7.46–10.49 ×10^6^ cells/mL at 7 dpi in the high-dose group and 8.50–11.23 ×10^6^ cells/mL at 9 dpi in the low-dose group, compared to pre-infection levels of 16.73–17.49 ×10^6^ cells/mL (Figure 4), while remaining stable in uninfected controls (8.70–10.11 ×10^6^ cells/mL). A significant reduction in lymphocyte counts (lymphopenia) was observed in both groups as infection progressed. In parallel, red blood cells (RBCs), hemoglobin (HGB), and platelet counts decreased after the early stages of infection, with earlier and more pronounced changes in the high-dose group. Overall, these findings indicate progressive leukopenia, particularly lymphopenia, accompanied by thrombocytopenia, with a more rapid onset in the high-dose group and a delayed but similar pattern in the low-dose group.

### 3.5. Biochemical Parameters

The functionality of the liver and kidney was examined by evaluating the level of alanine aminotransferase (ALT), aspartate aminotransferase (AST), and Albumin (ALB) for the liver, and the level of creatinine (CREA), and urea (UREA) for kidney in serum of pigs infected with recombinant ASFV genotype I and II in doses of 10^5^ HAD_50_/mL and 10^3^ HAD50/mL of NIVR-LS100. ALT levels were elevated and increased after the early stages of infection in both groups compared to the uninfected control groups. The highest level was 71.23 U/L in pig # 3 at 7 dpi of dosed 1 × 10^5^ HAD_50_/mL and 80.43 U/L in pig #3 at 8 dpi of dosed 1 × 10^3^ HAD_50_/mL, compared with 59.89–63.45 U/L in the uninfected control group (Supplementary Figure S2). The total ALB was slightly decreased in all the pigs infected with recombinant ASFV genotypes I and II, and was lowest before the pig died (Supplementary Figure S2). In contrast, the level of AST was increased after 1 dpi and was highest at 3–4 dpi in pigs infected with 10^5^ HAD_50_/mL, and at 3 dpi and highest at 5–6 dpi in pigs infected with 1 × 10^3^ HAD_50_/mL, and then decreased over time to die in both groups (Supplementary Figure S2). On the other hand, the level of Urea was dramatically increased after 2 dpi and highest at 6 dpi of pig infected with 1 × 10^5^ HAD_50_/mL, and after 4 dpi and highest at 8–9 dpi of pig infected with 1 × 10^3^ HAD_50_/mL (Supplementary Figure S2). Furthermore, the level of CREA was slightly increased on 4–5 dpi and 5–6 dpi in dosed 1 × 10^5^ HAD_50_/mL and 1 × 10^3^ HAD_50_/mL of NIVR-LS100, respectively, compared to uninfected control groups (Supplementary Figure S2). Overall, these biochemical changes indicate progressive hepatic and renal dysfunction, with a dose-dependent pattern characterized by earlier and more severe alterations in the high-dose group, consistent with the acute and severe course of ASFV infection.

### 3.6. The Expression of Pro-Inflammatory in Pig Infection with ASFV Recombinant Genotypes I and II

**Figure 5:**
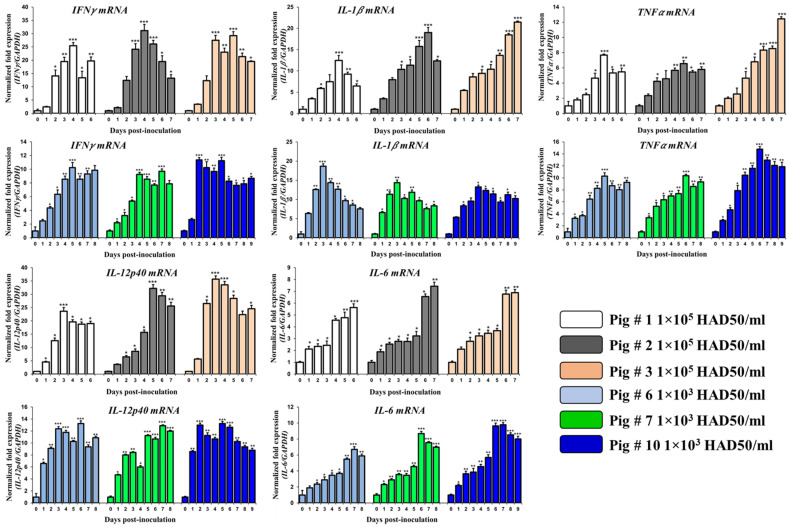
The expression of pro-inflammatory cytokines in whole blood cells. The expressions of IL-1β, TNF-α, IL-6, IFN-γ, and IL-12p40 in each pig were evaluated by quantitative RT-PCR. Individual data points are color-coded and presented as means (±SD); significant differences between pre- and post-treatment were evaluated using one-way analysis of variance (ANOVA) with *: *p* < 0.05, **: *p* < 0.01, and ***: *p* < 0.001.

A recent report indicated that the high expression of cytokine genes is a “cytokine storm” in pigs infected with virulent ASFV [22,23,24]. In this study, we analyzed the expression of some pro-inflammatory cytokines, including *IFN-γ*, *TNF-α*, *IL-1β*, *IL-6*, and *IL-12p40* mRNA in whole blood of pigs infected with recombinant ASFV genotype I and II in doses of 1 × 10^5^ HAD_50_/mL and 1 × 10^3^ HAD_50_/mL of NIVR-LS100. The results in Figure 5 showed that the expression of *IFN-γ* mRNA increased significantly and was highest after 3–4 dpi in pigs infected with 1 × 10^5^ HAD_50_/mL and after 4–5 dpi in pigs infected with 1 × 10^3^ HAD_50_/mL. With dose 1 × 10^5^ HAD_50_/mL, the highest expression of *IFN-γ* mRNA was evaluated as 31.17-fold change after 4 dpi in pig #2, and with dose 1 × 10^3^ HAD_50_/mL, the highest expression of *IFN-γ* mRNA was evaluated as 11.25-fold change after 5 dpi in pig #10 (Figure 5). *IL-1β* mRNA was strongly increased since 2 or 3 dpi, but showed slightly different patterns in the two infections.

With dose 1 × 10^5^ HAD_50_/mL, *IL-1β* mRNA levels of pigs #1 and #2 significantly increased and were highest at 4 dpi and 6 dpi as 12.45-fold change and 18–99-fold change, respectively, and then decreased over time to death. In contrast, the *IL-1β* mRNA expression of pig # 3 was dramatically increased and reached its highest level at 7 dpi with a 21.45-fold change (Figure 5). With a dose of 1 × 10^3^ HAD50/mL, *IL-1β* mRNA levels in pigs #6 and #7 increased significantly and reached their highest levels at 3 dpi, with 18.86-fold and 14.36-fold changes, respectively, and then decreased over time until death. In pig #10, *IL-1β* mRNA expression was significantly upregulated and reached its highest level at 4 dpi with a 13.25-fold change (Figure 5). Regarding *IL-12p40* mRNA, the level significantly started to rise at 2–4 dpi with a dose of 1 × 10^5^ HAD_50_/mL and 1–2 dpi with a dose of 1 × 10^3^ HAD_50_/mL (Figure 5). In contrast, the expression of *IL-6* mRNA was drastically increased in the terminal phase in pigs. This increase was also observed after 5 dpi in pigs infected with both doses (Figure 5). Like other pro-inflammatory cytokines, the expression of *TNF-α* mRNA also increased at 3 or 4 dpi. It was maintained at a high level until the terminals (Figure 5).

**Figure 6:**
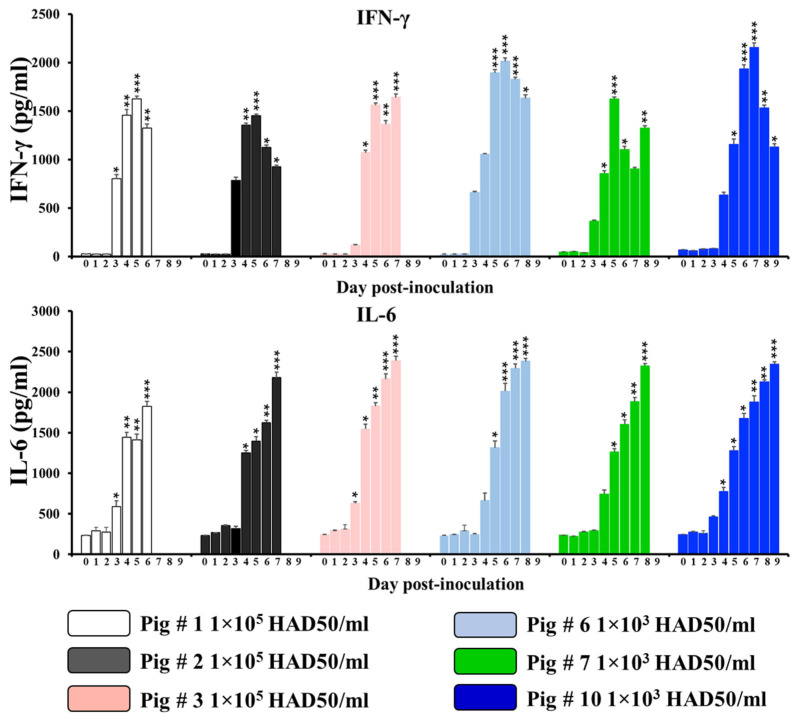
Kinetics of serum representative pro-inflammatory cytokines. The concentrations of interleukin (IL)-6 and IFN-γ in each pig were evaluated by quantitative ELISA. Individual data points are color-coded and presented as mean (±SD); significant differences between pre- and post-treatment were evaluated using one-way analysis of variance (ANOVA) with *: *p* < 0.05, **: *p* < 0.01, and ***: *p* < 0.001.

We continuously evaluated the kinetics of the most important pro-inflammatory cytokines—*IFN-γ* and *IL-6*—in the serum of pigs upon recombinant ASFV genotype I and II in doses of 1 × 10^5^ HAD_50_/mL and 1 × 10^3^ HAD_50_/mL of NIVR-LS100 infection. *IFN-γ* increased from 3 or 4 dpi but showed slightly different patterns at the two doses of infection. At a dose of 1 × 10^5^ HAD_50_/mL, the protein levels of IFN-γ in pigs #1 and #2 increased approximately 32 times, from 24 to 25 to 800 pg/mL at 3 dpi, whereas that of pigs #3 displayed an increase of approximately five times from 25.36 to 116.30 pg/mL at 3 dpi. On the other hand, at a dose of 1 × 10^3^ HAD50/mL of NIVR-LS100 infection, the IFN-γ protein level increased 25-fold at 3 dpi in pig #6, nine-fold in pig #7, and approximately eight-fold at 4 dpi in pig #10 (Figure 5). The concentration of IL-6 protein increased from an average of 313.27 to 511.65 pg/mL at 3 dpi and from 332.64 to 726.88 at 5 dpi at doses of 1 × 10^5^ HAD_50_/mL and 1 × 10^3^ HAD_50_/mL of ASFV NIVR-LS100 infection, respectively, and kept rising throughout the course of the disease (Figure 6). Results showed that ASFV recombinant genotype I and II infections induced an increase in the level of *IFN-γ*, *TNF-α*, *IL-1β*, *IL-6*, and *IL-12p40* mRNA in whole blood and increased levels of IFN-γ and IL-6 in serum.

### 3.7. The Chemokine Expression in Pig Infection with ASFV Recombinant Genotypes I and II

**Figure 7:**
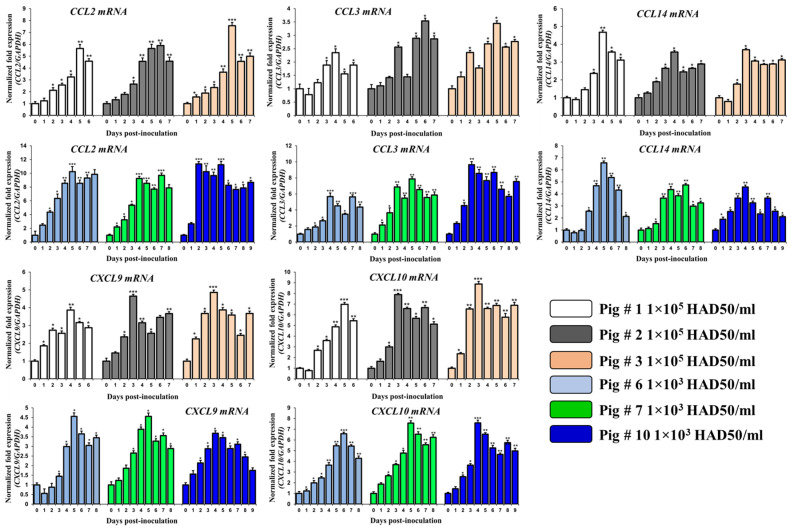
The expression of chemokines in whole blood cells. The expressions of CCL2, CCL3, CCL14, CXCL9, and CXCL10 in individual pigs were evaluated by quantitative RT-PCR. Individual data points are color-coded and presented as means (±SD); significant differences between pre- and post-treatment were evaluated using one-way analysis of variance (ANOVA) with *: *p* < 0.05, **: *p* < 0.01, and ***: *p* < 0.001.

Chemokine genes selected for analysis were inflammatory chemokines and known to be expressed in macrophages [24,25]. Changes in mRNA levels were measured relative to calibrator samples collected from pigs’ pre-infections. With dose 1 × 10^5^ HAD_50_/mL, after 1 dpi, mRNA levels for *CCL2*, *CCL3*, *CCL14*, *CXCL9*, and *CXCL10* were significantly increased in samples from infected pigs at all times compared to uninfected control pigs. The highest of *CCL2*, *CCL3*, *CCL14*, *CXCL9*, and *CXCL10* expressions were significantly upregulated at 5 dpi (7.56-fold change), 6 dpi (3.65-fold change), 4 dpi (4.68-fold change), 3 dpi (4.86-fold change), and 3 dpi (8.88-fold change) (Figure 7). On the other hand, the expressions of *CCL2*, *CCL3*, *CCL14*, *CXCL9*, and *CXCL10* in pigs infected with 1 × 10^3^ HAD_50_/mL were similarly upregulated compared to uninfected control pigs (Figure 6). Results showed that ASFV recombinant type I and II infections induced a rise in the level of *CCL2*, *CCL3*, *CCL14*, *CXCL9*, and *CXCL10* very early at 2 or 3 dpi, and this increasing tendency remained throughout the disease.

## 4. Discussion

In this study, we successfully isolated and characterized the highly lethal recombinant ASFVs from domestic pig farms in the provinces of North Vietnam. The ASFV isolates, NIVR-LS100, NIVR-LS102, and NIVR-LS08, were characterized by the HAD assay, real-time PCR, and sequencing analysis. The ASFV isolates replicated efficiently in primary PAM, and their viral titers reached 10^6.4^ to 10^7.4^ HAD_50_/mL. Phylogenetic analysis based on the p72 and p54 genes indicated that three viruses are highly similar to HeN/123014/22, IM/DQDM/22, and JS/LG/21, which caused the first ASFV recombinant genotype I and II in China, 2023 [17]. These results provide important information to help trace the source of these viruses through field surveillance and imply that the same events may have led to the emergence of these recombinant genotype I–II ASFVs in Vietnam.

Animal studies demonstrated that the ASFV NIVR-LS100 virus replicates systemically and is highly virulent in pigs. Recently, studies indicated that the highly virulent ASFV infected domestic pigs, and the pigs died starting after 3 dpi [26,27,28]. In our research, pigs inoculated with different dosages of NIVR-LS100 began to show early disease signs by 3–4 dpi with 1 × 10^5^ HAD_50_/mL and 4–5 dpi with 1 × 10^3^ HAD_50_/mL, and all of the animals died between 6 and 8 dpi with 1 × 10^5^ HAD_50_/mL and 8–10 dpi 1 × 10^3^ HAD_50_/mL, respectively, indicating that the ASFV strain NIVR-LS100 virus causes acute disease in domestic pigs. Of note, the disease signs and necropsy changes caused by ASFV are similar to those caused by previous studies, including Georgia 2007/1, Armenia 07, Pig/Heilongjiang/2018, Pig/HN/07, ASFV Mongolia/2019, Korea/Pig/Hongcheon/2022, and Korea/Pig/Pocheon/2023 [26,27,29,30,31,32]. All pigs inoculated with the recombinant ASFV strain NIVR-LS100 exhibited consistent clinical signs, including high fever, anorexia, fatigue, dyspnea, skin hemorrhaging, and gastrointestinal or respiratory symptoms. Mortality occurred between 6 and 8 dpi with a dose of 1 × 10^5^ HAD_50_/mL, and between 8 and 10 dpi with a dose of 1 × 10^3^ HAD_50_/mL. Respiratory distress, joint swelling, ocular discharge, melena, and epistaxis were also observed in pigs given the higher dose and, in some pigs, receiving the lower dose. At necropsy, all challenged pigs displayed pathological lesions such as enlarged hemorrhagic lymph nodes, including those in the intestine, groin, submaxillary area, bronchi, gastrohepatic region, mediastinum, and splenomegaly with infarction [1,26]. The virulence of ASFV strains can vary widely even within the same host. As of now, NIVR-LS100 is the recombinant ASF virus identified and tested in Vietnamese pigs, indicating the presence of a highly virulent strain within Vietnam’s pig population. Further isolation and analysis of additional viruses are necessary to fully comprehend the spread of disease and to develop effective control strategies.

Variations in clinical presentations among individual pigs stem from differences in virulence and interactions between the host and virus [33]. These findings hold significant educational value for farmers, veterinary officers, and clinical veterinarians. Viremia was detectable at 3–4 dpi, followed by viral shedding via oral, nasal, and rectal routes, concurrent with observable clinical signs. The viral genome persisted from initial detection until death across all infected pigs, albeit with varying viral loads. Notably, EDTA-treated whole blood exhibited the highest viral load compared to swab samples. Although nasal and rectal swabs showed increased viral loads in pigs displaying signs such as epistaxis and melena, these did not exceed those found in whole blood. While swab samples offer an alternative, their delayed detection by 1–2 days compared to whole blood may result in missed diagnoses during the incubation period. Given the periodic nature of veterinary surveillance, this delay heightens the risk of misdiagnosis and the potential for broader outbreaks. Therefore, whole blood remains the preferred and reliable sample type in regions where highly virulent ASFV strains are circulating [33]. In deceased pigs, ASFV genomes were detected in all seven organs collected during necropsy. The spleen, liver, and lymph nodes exhibited the highest viral loads compared to other organs, illustrating variability in tissue distribution influenced by virus strain, infection dose, route, and virus–host interactions.

The white blood cells (WBCs), red blood cells (RBCs), hemoglobin levels, lymphocytes, and platelet cells are important components of the animal’s immune system [34]. The WBC count is a powerful indicator for infectious and inflammatory diseases such as leukemia, lymphoma, and bone marrow disorders. The RBC parameters are important innate immune cells in the blood circulation, and they can recognize and kill antigens; they are also involved in immune regulation and have a complete self-regulation system [35,36]. On the other hand, a lymphocyte is a type of white blood cell that is part of the immune system. There are two main types of lymphocytes: B cells and T cells. B cells produce antibodies that attack invading bacteria, viruses, and toxins. The T cells destroy the body’s own cells that have been taken over by viruses or become cancerous [34,35,36,37]. Recent studies of ASFV genotype II and recombinant genotype I–II in Asia strain showed that WBC counts in ASFV-infected pigs dramatically increased in early infection and decreased over time as the pig died [15,16,27,31,38,39]. Our study indicated that the increase in WBC counts was observed in early infection (until 3–4 dpi) in both groups and then decreased in ASFV-infected pigs until the last day of life. These results suggest that, compared to the Asian genotype II and recombinant genotype I–II strains, the Vietnamese strain showed similar patterns of changes in WBC counts in ASFV-infected pigs. Recently, research indicated that hemorrhage in the skin and organs of pigs infected with ASFV is associated with increased vascular permeability and reductions in RBC and hemoglobin [37,40]. Our results have shown a decrease in RBC counts, hemoglobin levels, and platelet count after early infection (after 3–4 dpi), which is consistent with the previous study.

The reduction in lymphocytes was observed in both groups 3 days before death, as previously reported in pigs infected with high-dose ASFV genotypes from Vietnam, Korea, and China [27,31,38,39,41,42,43]. Furthermore, serum biochemical profiles were observed in pigs infected with recombinant ASFV genotype I–II and focused on the liver and kidney functions, such as ALT, Albumin, AST, Uric acid, and Creatine levels. Our results indicated an increase in the level of ALT, Uric acid, and creatine in pigs infected with recombinant ASFV after 2–3 dpi with 1 × 10^5^ HAD_50_/mL and 3–4 dpi with 1 × 10^3^ HAD_50_/mL. In contrast, a decrease in albumin level was observed in pigs infected with recombinant ASFV after 3–4 dpi with 1 × 10^5^ HAD_50_/mL and 6–7 dpi with 1 × 10^3^ HAD_50_/mL. On the other hand, the increase in AST level after 1 dpi with 1 × 10^5^ HAD_50_/mL and 3 dpi with 1 × 10^3^ HAD_50_/mL, and then the diseases after 3–4 dpi and 5–6 dpi in doses 1 × 10^5^ HAD_50_/mL and 1 × 10^3^ HAD_50_/mL, respectively. Our results were consistent with the previous study on the effect of genotype II ASFV, a highly pathogenic strain circulating in pigs in Asian countries [27,31,38,39,40,41,42,43,44]. The results of the present study support that the disease causes alterations in the blood and serum biochemical parameters in the infected pigs. The disease is associated with significantly lower RBC count, WBC count, absolute lymphocyte count, absolute platelet count, serum total albumin, AST, and hemoglobin levels, and significantly higher ALT, uric acid, and creatinine levels. Knowledge of the blood–biochemical changes may be useful to predict the prognosis of the disease. Veterinarians should consider the blood and biochemical profiles of ASF to formulate effective supportive therapy to ameliorate the morbidity and high case fatality caused by the disease.

Whole blood contains various immune cells, including mononuclear (T cells, B cells, NK cells, monocytes–macrophages) and polymorphonuclear cells (granulocytes), which play an important immune system and a significant role in the body’s immune response, orchestrating defense mechanisms against infections and diseases [35,36,37]. Furthermore, several studies indicated that the primary monocyte–macrophage cells played an important role in the replication of ASFV and induced the expression of cytokines, chemokines, and immune-related genes [45,46,47]. Recent reports demonstrated that the low-virulent and virulent ASFV induced the expression of several cytokines and chemokines in vitro and in vivo, such as IFN-α, IFN-β, IFN-γ, IL-18, IL-1β, IL-1α, IL-6, IL-8, TNF-α, IL-12, CCL2, CCL4, CXCL9, and CXCL10 [23,24,48,49,50,51]. Based on cytokine expression profiles in whole blood and the levels of IFN-γ and IL-6 in serum from both the present and previous studies, the course of acute infection with highly virulent recombinant ASFV genotypes I–II can be divided into two phases. The first, or primary phase, spans 0–1 dpi and is characterized by minimal or no changes in cytokine expression in whole blood, accompanied by an absence of clinical signs in pigs infected with the virulent recombinant ASFV genotype I–II [23]. The second phase is the clinical phase, where pigs infected with a virulent recombinant genotype I–II have shown clinical symptoms and upregulated pro-inflammatory cytokine and chemokines in whole blood and sustained fever from 2 to 7 dpi. Recently, research suggested that type I IFNs such as IFN-α, IFN-γ, or IFN-β have been playing an important role in the immune response to ASFV infection in vitro and in vivo because ASFV genomes such as MGF360, MGF505, pI329L, pA238L, and pDP96R interfered with the induction of the IFN-α, IFN-γ, or IFN-β pathway, and modulated the NF-kB signaling pathway, cGAS-STING signaling pathway, or JAK/STAT signaling pathway, leading to induced expression of the cytokines, chemokines, and immune-related genes [24,51,52,53]. This could explain the unchanged levels of type I IFN and other pro-inflammatory cytokines during the primary phase of infection with a virulent recombinant ASFV genotype I–II. Due to the incapability of type I IFN induction by a virulent recombinant ASFV genotype I–II in whole blood, the dramatic elevation of IFN-γ during the clinical phase of infection could be a result of secondary activation of immune cells in whole blood [23]. Our data strongly suggest that pig infection with a virulent recombinant ASFV genotype I–II induced cytokines and chemokine expression, and provides further insights into the immune evasion strategies of ASFV, which could facilitate the development of antiviral drugs and novel theoretical frameworks for vaccine development using gene deletion, subunit, or viral vector methods.

## 5. Conclusions

In summary, we report that recombinant genotype I–II ASFVs have also emerged in Vietnam. Animal challenge revealed that recombinant genotype I–II ASFV NIVR-LS100 showed high virulence and was found to cause necrotic skin lesions and joint swelling. The blood cell count dynamics and serum biochemistry profiles were similar between the high and low doses of ASFV infection; however, viral load distribution was different. Furthermore, analysis of whole blood demonstrated a marked and sustained increase in pro-inflammatory cytokines and chemokines, including TNF-α, IFN-γ, IL-1β, IL-6, IL-12p40, CCL2, CCL3, CCL14, CXCL9, and CXCL10, coinciding with persistent fever from 2 to 7 dpi. This cytokine and chemokine profile is indicative of a classical cytokine storm, characterized by delayed and dysregulated secretion of pro-inflammatory mediators and an imbalance between pro- and anti-inflammatory responses. These findings provide deeper insight into the molecular mechanisms underlying ASFV pathogenesis. Consequently, the emergence of recombinant genotype I–II ASFVs in Vietnam presents significant challenges for ASF eradication efforts. These newly emerging recombinant genotypes I–II ASFVs could cause severe and ongoing economic losses to the pig industry if they spread within swine herds or infect breeding sows and boars. Therefore, nationwide surveillance of recombinant genotype I–II ASFVs is urgently needed to minimize the impact of these infections in Vietnam.

Limitations of this study: This study has several limitations that should be considered when interpreting the findings. First, histopathological and immunohistochemical analyses were not conducted, limiting detailed assessment of microscopic lesions, tissue tropism, and cellular targets associated with infection. Second, detailed genomic characterization of the recombinant ASFV strain was limited; although sequencing and phylogenetic analyses support its classification as a genotype I/II recombinant, a comprehensive recombination map and gene-level analysis were not performed. In addition, the lack of direct comparative infections with classical genotype I and genotype II reference strains remains a major limitation, as it restricts precise evaluation of the relative virulence and biological characteristics of the NIVR-LS100 strain. Third, viral detection was based on qPCR without absolute quantification or standard curve calibration, and serological analysis was not included due to the acute and rapidly fatal disease course. Finally, in vitro characterization and vaccine-challenge studies were not performed, limiting the interpretation of viral replication dynamics and implications for vaccine efficacy. Despite these limitations, the study provides a comprehensive in vivo characterization of a naturally occurring recombinant ASFV strain and demonstrates a highly virulent phenotype with potential implications for ASF control and surveillance.

## Figures and Tables

**Table 1:** Sequences of oligonucleotide primers for real-time RT-PCR.

Genes	Oligonucleotide Sequences (5′–3′)	GenBank Acc.No
GAPDH	F: ACACTCACTCTTCTACCTTTG	NM_001206359.1
	R: CAAATTCATTGTCGTACCAG	
IFNγ	F: CAGCTTTGCGTGACTTTGTG	X53085
	R: GATGAGTTCACTGATGGCTTT	
IFNα	F: CCCCTGTGCCTGGGAGAT	XM_003480507.1
	R: AGGTTTCTGGAGGAAGAGAAGGA	
IFNβ	F: AGTTGCCTGGGACTCCTCAA	GQ415073.1
	R: CCTCAGGGACCTCAAAGTTCAT	
TNFα	F: CGTTGTAGCCAATGTCAAAGCC	X54859
	R: TGCCCAGATTCAGCAAAGTCCA	
IL-1α	GTGCTCAAAACGAAGACGAACC	NM_214029.1
	CATATTGCCATGCTTTTCCCAGAA	
IL-1β	F: TGAAGAGAGAAGTGGTGTTCTGC	AJ747049
	R: GGTACAGATTCTTTCCCTTGATCC	
IL-12p40	F: GATGCTGGCCAGTACACC	U08317
	R: TCCAGCACGACCTCAATG	
IL-17A	F: CAAGCGGTGGCGTTTTGCCT	NM_001005729.1
	R: GTCTCCGTCGGGGATGGGCT	
IL4	F: ATCCCAACCCTGGTCTGC	X68330
	R: TCCTGTCAAGTCCGCTCA	
IL-10	F: GCCTTCGGCCCAGTGAA	NM_214041.1
	R: AGAGACCCGGTCAGCAACAA	
IL-11	F: CCGCACAGCTGAGAGACAAAT	XM_021095008.1
	R: GCCTCAGGTAGGAAAACAGGT	
IL-13	F: GGCAGTTTTCCTGCTTTCT	NM_213803.1
	R: GGCAGTTTTCCTGCTTTCT	
CCL-2	R: GCGGCTGATGAGCTACAGAAG	NM_214214
	R: CCGCGATGGTCTTGAAGATC	
CCL-3	R: CTTCCTCGCAAATTCGTAGC	NM_001009579
	R: GCATTCAGCTCCAGGTCAG	
CCL-14	R: ACCCAGCACTTTTACCGAGG	NM_001256775
	R: TGTCTGGAGCGCAAGAGAAG	
CXCL-9	R: CTCAGCTTTTCCCGCAGAGT	NM_001114289
	R: TTGGTGGCCTTCTTGTCAGG	

## Data Availability

The original contributions presented in the study are included in the article. Further inquiries can be directed to the corresponding authors.

## Supplementary Materials

The following supporting information can be downloaded at: https://www.mdpi.com/article/10.3390/v18060635/s1, Table S1: Clinical signs used to calculate African swine fever clinical sign scores. The list was generated based on the protocol published by Galindo-Cardiel et al., Table S2: Gross organ lesion; Figure S1. Gross lesions in pigs that died following infection with African swine fever virus (ASFV) NIVR-LS100 (10^5^ HAD_50_/mL). Representative lesions are shown from the skin and major organs. (A,B) Skin: multifocal petechiae and ecchymoses with areas of diffuse cutaneous congestion. (C,D) Mandibular and superficial inguinal lymph nodes: markedly enlarged, edematous, and dark red, consistent with severe congestion and hemorrhage. (E) Heart: epicardial petechiation with mild to moderate edema along the coronary/interventricular grooves. (F) Lung: diffuse pulmonary congestion and edema with multifocal hemorrhagic areas. (G) Spleen: splenomegaly with dark red to blackish discoloration, friable parenchyma consistent with severe congestion. (H,I) Mesenteric lymph nodes and intestinal serosa: enlarged lymph nodes with marked congestion/hemorrhage; intestinal serosa showing congestion with scattered petechiae. (J) Kidney: cortical congestion with occasional petechiae. (K) Stomach: mucosal and serosal congestion with multifocal petechiae and ecchymoses. (L) Testicles: enlarged and congested with dark red discoloration. Figure S2: Biochemical parameters of the liver and kidneys. ALT, alanine aminotransferase; AST, aspartate aminotransferase; CREA, creatinine; UREA, urea; and ALB, Albumin. The reference intervals of each blood parameter are represented as dotted lines.

## Abbreviations

The following abbreviations are used in this manuscript:

ASFV	African swine fever virus
HAD	Haemadsorption Dose
PAMs	Porcine alveolar macrophages
PBMCs	Peripheral blood mononuclear cells
RT-qPCR	Quantitative real-time reverse transcription PCR
ELISA	Enzyme-linked immunosorbent assay
RPMI	Roswell Park Memorial Institute
SVN	Serum virus-neutralizing
TNF-α	Tumor necrosis factor-alpha
mAbs	Monoclonal antibodies
RBCs	Red blood cells
TCID	Tissue culture infective dose
IFN	Interferon
IL	Interleukin
MHC	Major histocompatibility complex
CPE	Cytopathic effect
DMEM	Dulbecco’s modified Eagle’s medium
Dpc	Days post-challenge
FBS	Fetal bovine serum
ANOVA	Analysis of variance
SEM	Standard error of the mean
CCL	Chemokine (C-C motif) ligand
CXCR	C-X-C chemokine receptor
