# Supplementary material for: Evaluation of Pathogenetic and Immunological Parameters of the Genotype I and II Recombinant African Swine Fever Viruses Detected in Pigs in Vietnam
Source: Viruses. 2026 May 31;18(6):635. doi: 10.3390/v18060635 (PMC13307865; doi:10.3390/v18060635)
Supplement: Supplementary file 1 [file viruses-18-00635-s001.zip › viruses-4275422-supplementary.pdf]

Supplement data:

Table S1. Clinical signs used to calculate African swine fever clinical sign scores. The list was generated based on the protocol published by Galindo-Cardiel et al.

Clinical signs	Scores (0: normal, 1: mild, 2: moderate, and 3: severe)
Fever	0: <39.5°C<br>1: 39.5–40.5 °C<br>2: 40.6–41.0 °C<br>3: > 41.0 °C
Behavior	0: Normal<br>1: Decreased activity, mild to moderate clumsiness<br>2: Decreased external stimuli response<br>3: Markedly decreased or lack of external stimuli response, immobile, prostration
Skin	0: Normal<br>1: Body cyanotic areas (< 10%); minimal multifocal cutaneous necrosis and/or hemorrhages<br>2: Body cyanotic areas (11-25%); mild to moderate multifocal cutaneous necrosis and/or hemorrhages<br>3: Body cyanotic areas (> 25%); moderate to marked multifocal cutaneous necrosis and/or hemorrhages
Digestive system	0: Normal<br>1: Feces around the anus (mild diarrhea)<br>2: Feces covering posterior gluteus (moderate diarrhea)<br>3: Feces covering posterior gluteus with blood or extensive mucus (severe bloody diarrhea)
Respiratory system	0: Normal<br>1: Mild dyspnea (labored respiration)<br>2: Moderate dyspnea or cough<br>3: Marked dyspnea or severe labored respiration
Body condition	0: Normal, full stomach<br>1: Empty stomach, sunken flanks<br>2: Empty stomach, sunken flanks, loss of muscle mass<br>3: Emaciated
**Total**	**From 0 to 18 (> 3 scores considered as the pigs showing clinical signs)**

Table S2. Gross organ lesion

Organ	Gross lesion	Group experiment

		10^5TCID50/ml	10^3TCID50/ml	Control
Spleen	Swelling	5/5 (100%)	5/5 (100%)	0/5 (0%)
	Congestion	5/5 (100%)	5/5 (100%)	0/5 (0%)
	Hemorrhage	5/5 (100%)	5/5 (100%)	0/5 (0%)
	Necrosis	5/5 (100%)	5/5 (100%)	0/5 (0%)
Lymph nodes	Swelling	5/5 (100%)	5/5 (100%)	0/5 (0%)
	Hemorrhage	5/5 (100%)	5/5 (100%)	0/5 (0%)
Tonsils	Swelling	5/5 (100%)	5/5 (100%)	0/5 (0%)
	Hemorrhage	5/5 (100%)	5/5 (100%)	0/5 (0%)
Liver	Swelling	5/5 (100%)	5/5 (100%)	0/5 (0%)
	Hemorrhage	4/5 (80%)	2/5 (40%)	0/5 (0%)
Lungs	Swelling	3/5 (60%)	2/5 (40%)	0/5 (0%)
	Hemorrhage	5/5 (100%)	3/5 (60%)	0/5 (0%)
	Pneumonia	2/5 (40%)	1/5 (20%)	0/5 (0%)
Kidneys	Swelling	4/5 (80%)	2/5 (40%)	0/5 (0%)
	Hemorrhage	2/5 (40%)	1/5 (20%)	0/5 (0%)
Heart	Hemorrhage	4/5 (80%)	2/5 (40%)	0/5 (0%)
	Pericardia fluid	5/5 (100%)	4/5 (80%)	0/5 (0%)
Intestinal	Hemorrhage	5/5 (100%)	4/5 (80%)	0/5 (0%)
Stomach	Hemorrhage	4/5 (80%)	2/5 (40%)	0/5 (0%)
Testicles	Swelling	3/3 (100%)	1/3 (33.33%)	0/3 (0%)
	Hemorrhage	3/3 (100%)	1/3 (33.33%)	0/3 (0%)
	Congestion	1/3 (33.33%)	1/3 (33.33%)	0/3 (0%)

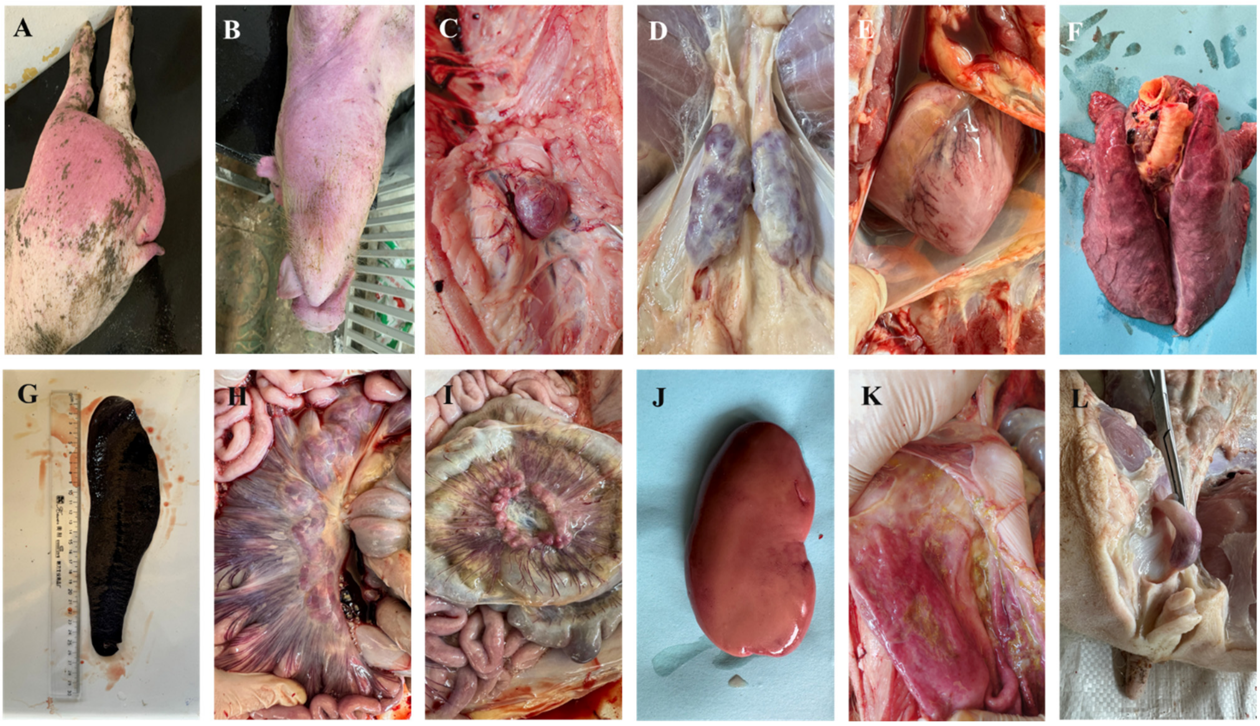

Figure S1. Gross lesions in pigs that died following infection with African swine fever virus (ASFV) NIVR-LS100 (10^5 HAD_{50}/mL). Representative lesions are shown from the skin and major organs. (A–B) Skin: multifocal petechiae and ecchymoses with areas of diffuse cutaneous congestion. (C–D) Mandibular and superficial inguinal lymph nodes: markedly enlarged, edematous, and dark red, consistent with severe congestion and haemorrhage. (E) Heart: epicardial petechiation with mild to moderate edema along the coronary/interventricular grooves. (F) Lung: diffuse pulmonary congestion and edema with multifocal hemorrhagic areas. (G) Spleen: splenomegaly with dark red to blackish discolouration, friable parenchyma consistent with severe congestion. (H–I) Mesenteric lymph nodes and intestinal serosa: enlarged lymph nodes with marked congestion/hemorrhage; intestinal se-rosa showing congestion with scattered petechiae. (J) Kidney: cortical congestion with occasional petechiae. (K) Stomach: mucosal and serosal congestion with multifocal pe-techiae and ecchymoses. (L) Testicles: enlarged and congested with dark red discolouration.

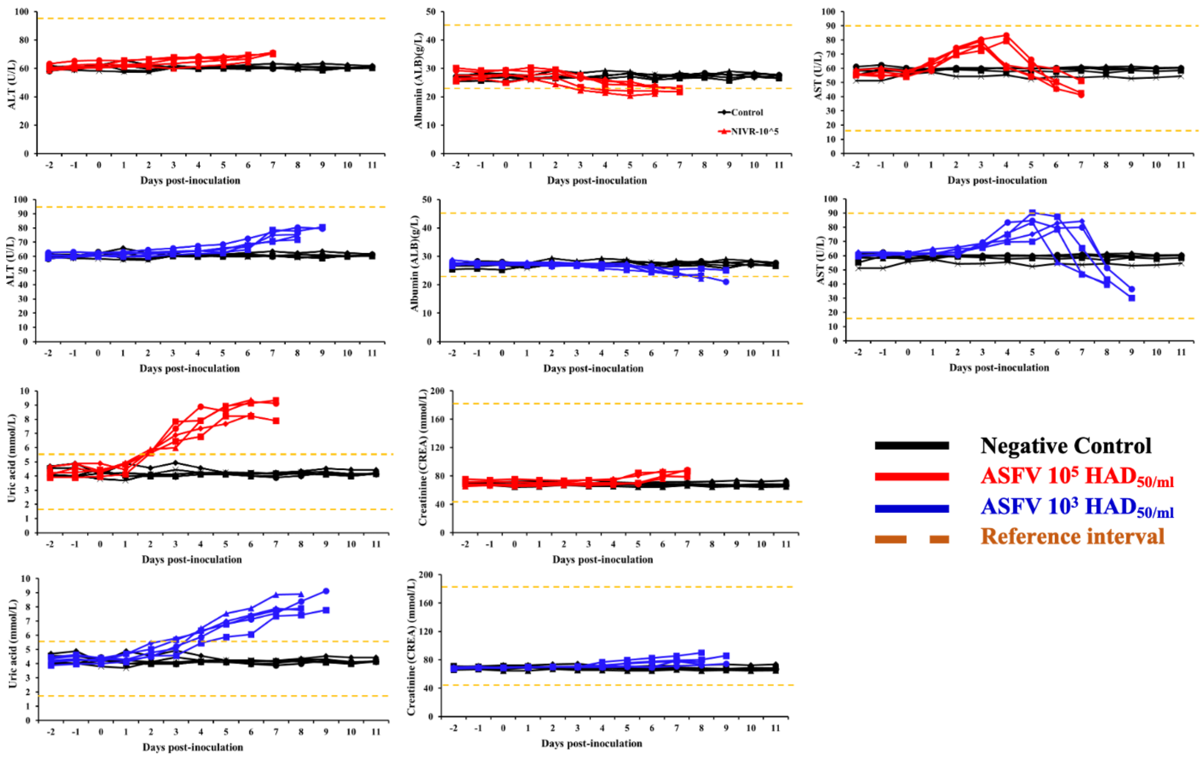

Figure S2. Biochemical parameters of the liver and kidneys. ALT, alanine aminotransferase; AST, aspartate aminotransferase; CREA, creatinine; UREA, urea and ALB, Albumin. The reference intervals of each blood parameter are represented as dotted lines.
